# A single-center experience of non-bioartificial DFAPP support systems among Chinese patients with hyperlipidemic moderate/severe acute pancreatitis

**DOI:** 10.1038/s41598-024-51761-w

**Published:** 2024-01-11

**Authors:** Xianwen Cheng, Yanrong Zhan, Zhendong Wang, Feng Wang, Xia Zeng, Ya Mao, YaoShun Liu

**Affiliations:** 1 Ankang Hospital of Traditional Chinese Medicine, Ankang, 725000 Shaanxi China; 2 Shaanxi University of Chinese Medicine, Xianyang, 712000 Shaanxi China

**Keywords:** Diseases, Gastrointestinal diseases, Pancreatic disease

## Abstract

To assess the clinical efficacy of Double Filtration Plasmapheresis (DFAPP), a novel blood purification method, in treating hyperlipidemic moderate/severe pancreatitis (HL-M/SAP). A total of 68 HL-M/SAP patients were enrolled in this study. The observation group, comprising 34 patients, received DFAPP treatment, while the control group underwent CVVH + PA treatment. We compared the efficacy changes between the two groups post-treatment. Patients treated with DFAPP showed significant improvements in clinical outcomes. After 72 h of DFAPP treatment, HL-M/SAP patients exhibited notably lower multiple organ failure scores and a reduced mortality rate compared to those in the CVVH + PA group. Triglyceride levels in HL-M/SAP patients treated with DFAPP for 48 h averaged 3.75 ± 1.95, significantly lower than the 9.57 ± 3.84 levels in the CVVH + PA group (P < 0.05). Moreover, CRP levels decreased markedly, IL-17 levels diminished, IL-10 levels increased, and the decline in IL-35 levels was significantly less pronounced compared to the CVVH + PA group. The recurrence rate of pancreatitis was also significantly lower after 6 months. The early implementation of DFAPP in HL-M/SAP patients effectively reduces triglyceride levels, suppresses pro-inflammatory factors, enhances anti-inflammatory factors, and mitigates cytokine storm-induced sepsis damage. Consequently, this leads to a decrease in the incidence of multiple organ failure, improved patient survival rates, and a reduce the recurrence rate of lipogenic pancreatitis.

**Trial registration:** Chinese Clinical Trial Registry, ChiCTR2300076066.

## Introduction

Moderate/Severe Acute Pancreatitis (M/SAP) is a severe digestive system disorder marked by pancreatic tissue edema, bleeding, and necrosis, resulting from autodigestion, often accompanied by serious complications including systemic inflammatory response syndrome and multi-organ dysfunction. Hypertriglyceridemia (HTG) has emerged as a principal risk factor for M/SAP, due to a variety of causes, and accounts for over 10% of all SAP instances^1–4^. HL-M/SAP, induced by high triglyceride levels, predominantly affects younger individuals and has a rapid, severe progression, characterized by an approximate 40% mortality rate and an annual recurrence exceeding 50%^5,6^. The primary approach to HL-M/SAP treatment combines multidisciplinary comprehensive therapies and selective blood purification techniques. Globally, therapies often encompass plasma exchange along with hemodialysis, while in China, strategies such as double plasma exchange, continuous renal replacement therapy (CRRT), and continuous plasma filtration adsorption (CPFA) are utilized, either singly or in succession. The pathogenesis of HL-SAP primarily involves lipotoxicity due to hypertriglyceridemia and cytokine storm sepsis (CSS). Based on the existing research by scholars both domestically and internationally, the authors innovatively combined two blood purification modes—plasma adsorption and double plasma exchange—to create the Double Filtration Plasmapheresis (DFAPP). This has been applied clinically for 3 years, achieving significant therapeutic effects, and it was patented in China, In 2021 (patent number: 202110671354.9). This report aims to provide guidance for clinical practice.

## Materials and methods

### General information

This research enrolled 68 HL-M/SAP patients admitted to our hospital between May 2019 and May 2022. Utilizing a random number table, participants were equally divided into the control group (34 cases) and the observation group (34 cases). Before treatment began, informed consent was secured from all participants, and the investigation adhered to the national Good Clinical Practice (GCP) guidelines. The study was registered at the Chinese Clinical Trial Registry (ChiCTR2300076066, date of registration: 22/09/2023), and received approval from the Ethics Committee for Biomedical Research Involving Human Subjects of Ankang City Traditional Chinese Medicine Hospital (Ethics Approval Number AKZYLL-KS001). The observation group consisted 20 males and 14 females (age range 28–56 years; mean age 36.5 ± 10.5 years) and had symptom onset to hospital admission times ranging from 3 to 25 h, averaging 11.0 ± 6.5 h. The control group included 18 males and 16 females (age range 32–56 years; mean age 38.5 ± 9.5 years), with a symptom onset to hospital admission duration ranging from 4 to 28 h, averaging 10.0 ± 5.5 h. A comparative analysis of baseline characteristics like gender, age, and time from symptom onset to admission revealed no significant disparities between the two groups (P > 0.05).

### Diagnostic criteria

The selection criteria for this study were outlined in the 2019 edition of the “Chinese Guidelines for the Diagnosis and Treatment of Acute Pancreatitis” published by the Pancreatic Disease Group of the Chinese Society of Digestive Diseases and the Editorial Board of the “Chinese Journal of Digestion”^6^. Eligibility was determined based on the following criteria: (1) the onset of acute, severe upper abdominal pain, possibly radiating to the back; (2) serum amylase and/or lipase levels exceeding thrice the upper normal limit; (3) CT/MRI revealing characteristic AP imaging changes such as pancreatic edema or peripancreatic fluid collection. A clinical diagnosis required meeting two out of the three criteria, leading to an AP diagnosis. Furthermore, an M/SAP classification indicated persistent organ dysfunction or failure lasting between 24 and 48 h, accompanied by a Modified Marshall Score ≥ 2 points.

### Inclusion criteria

The study included patients who had provided signed informed consent, satisfied the criteria outlined in the 2019 “Chinese Guidelines for the Diagnosis and Treatment of Acute Pancreatitis” for SAP/MSAP, and exhibited triglyceride levels ≥ 11.3 mmol/L.

### Exclusion criteria

Exclusion from the study applied to individuals with coronary artery atherosclerotic heart disease classified as III-IV according to the New York Heart Association (NYHA), as well as those with malignancies, recent cardiovascular or cerebrovascular incidents, chronic renal failure, pregnancy or breastfeeding, mental disorders, or those over the age of 79 years. Further exclusion factors encompassed severe active bleeding, disseminated intravascular coagulation, allergies to treatment medications such as heparin or fish protein, circulatory failure, instability during myocardial infarction phases, and advanced pregnancy stages.

### Adverse event monitoring and management

Adverse reactions were meticulously recorded, detailing the timing, vital signs, clinical manifestations, laboratory tests, and management strategies at baseline, and on days 1 and 7 post-treatment. In complications arose during surgery—including bleeding, clotting, thrombosis, infection, allergic reactions, or imbalance syndrome—prompt interventions were initiated. Blood purification-related adverse events activated emergency protocols. In cases of severe adverse events, surgical procedures were halted, prioritizing life-saving measures and facilitating the transfer of patients to the ICU or appropriate departments for urgent rescue and treatment.

### Exclusions and withdrawals

Participants who couldn’t adhere to the prescribed treatment plan, or those who didn’t attend regular follow-up sessions, resulting in data gaps that compromised the evaluation of effectiveness and safety, were considered for removal from the study. Similarly, those who withdrew during the observation timeframe, ingested substances that potentially skewed the study outcomes, or encountered severe adverse events, complications, or allergies to the investigational drug, were deemed unfit for continued participation and could voluntarily exit the study.

## Research methodology

### Participant segmentation

Utilizing a prospective, randomized controlled approach, this study ensured that all participants meeting the inclusion criteria exhibited uniform baseline characteristics such as gender, age, and illness duration. Subsequently, they were evenly distributed into two groups: the observation group and the control group, each comprising 34 individuals.

### Intervention approaches

All participants underwent comprehensive standard internal medicine treatment, primarily focusing on acid suppression, enzyme inhibition, and fluid replenishment. The observation group received additional DFAPP treatment, while the control group was administered Continuous Veno-Venous Hemofiltration plus Plasma Adsorption (CVVH + PA) therapy, utilizing the Japanese JUN-55X blood purification treatment machine. Initial treatment involved the insertion of a single-lumen double-cavity catheter (Abel 11.5 cm × 13.5 cm) in the femoral vein. (1) DFAPP Mode: The treatment began with plasma separation utilizing the Japanese Asahi Kasei membrane-type plasma separator OP-08 (Lot: AG8Q96), followed by a secondary phase of filtration and adsorption of high-molecular-weight substances such as triglycerides, inflammatory factors, and endotoxins using the HA330-II resin hemoperfusion device (Zhuhai LiZhu Medical Biologic Materials Co., Ltd.). Each session lasted between 3 and 4 h, involving approximately 5 L of plasma replacement and adsorption. Typically, patients in this group underwent a single session of this treatment. (2) CVVH + PA Mode: This approach paralleled the DFAPP method, commencing with plasma separation using the aforementioned Japanese Asahi Kasei separator and the HA330-II resin hemoperfusion device for the absorption of plasma inflammatory factors. This phase lasted 3–4 h, facilitating around 5 L of plasma replacement and adsorption. Subsequently, patients received a combination of CVVH treatments extending for 6–8 h. Generally, individuals in this category experienced 1–2 treatment sessions. To mitigate the risk of allergic reactions, prophylactic measures included the administration of intravenous dexamethasone (H20130301, 5 mg, Pfizer Belgium) combined with 20 mL of 5% calcium gluconate prior to the treatment. Additionally, a low-molecular-weight heparin sodium injection (H31022051, 4000U, Shanghai First Biochemical Pharmaceutical Co., Ltd.) served as an anticoagulant, with dosages being fine-tuned based on individual coagulation profiles. Throughout the process, meticulous monitoring of various parameters including electrocardiography, blood pressure, and oxygen saturation was conducted, ensuring a comprehensive oversight of any changes in the patient's status.

### Observed indicators

#### Biochemical indicators

Before blood purification treatment, the subsequent morning, and on the morning of discharge, blood samples were collected from patients. These samples were analyzed using automated biochemical (Catalog No. E1006) and coagulation (CS5100, Sysmex Corporation, Japan) analyzers to assess various parameters including lipid profiles (triglycerides, cholesterol, high-density lipoprotein, low-density lipoprotein), procalcitonin (PCT), C-reactive protein (CRP), complete blood count, liver function tests, and coagulation function, among others.

#### Cytokines

The detection of cytokines IL-6, TNF-α, IL-17, IL-10, and IL-35 was conducted using the ELISA method. Initially, 5 mL of venous blood was collected into vacuum tubes fortified with EDTA anticoagulant for the analysis of IL-35, IL-10, IL-6, and TNF-α. These samples were refrigerated at 4 °C for 4 h before undergoing serum separation through a 5-min centrifugation process at 3000 rpm. Subsequently, the serum was extracted and stored at − 80 °C pending further analysis. Utilizing the Ang-2 assay kit from EasyMer (Beijing) Biotechnology Co., Ltd., and the MK3 enzyme immunoassay instrument from Thermo Fisher Scientific Inc. (USA), the standard protocol outlined in the sandwich ELISA assay kit was followed meticulously. Specifically, 100 μL of both standard solutions and test samples were added into the reaction wells and incubated at 37 °C for a span of 2 h. Following an initial wash, biotin-labelled enzyme reagents were introduced and incubated at 37 °C for an hour. This was succeeded by the addition of HRP-labelled streptavidin and a further 45-min incubation at 37 °C. Subsequently, the chromogenic solution was introduced, catalyzing a 30-min reaction at ambient temperature. This procedure culminated in the termination of the reaction, with the absorbance documented at a 450 nm wavelength. Concentrations were ascertained through the extrapolation of absorbance values mapped on the standard curve. The cytokine levels of IL-35, IL-10, and others were gauged across the MAP group, SAP group, and the normal control group. For IL-17 analysis, 10 mL of peripheral venous blood, treated with heparin anticoagulant, was procured from the selected study participants. PBMCs were segregated utilizing the Ficoll density gradient centrifugation method and maintained at a concentration of 2 × 10^6^/mL in RPMI-1640 medium, inclusive of 10% fetal bovine serum, 100 U/mL penicillin, and 0.1 ng/mL streptomycin. Thereafter, PBMC suspension (2 × 10^6^/mL) allocated into 24-well culture plates (1 mL per well), supplemented with PMA (20 ng/mL) and ionomycin (1 µg/mL), and incubated for 2 h in a culture incubator set at 37 °C and 5% CO_2_. Following the addition and subsequent two-hour incubation with monensin (2 nmol/mL), cells were harvested and centrifuged at 1500 rpm for 5 min, then rinsed with 2 mL of PBS. The subsequent procedure involved the partitioning of cells into control and experimental tubes, where they underwent a series of incubations and washes in preparation for analysis through flow cytometry. This comprehensive procedure ensures accurate and reliable data collection for the ongoing study.

#### Evaluation criteria

The APACHE II score, a prominent tool for gauging acute physiology and chronic health evaluations, was leveraged to scrutinize the patients' physiological condition, holding a pinnacle score of 71 points. Furthermore, the dual-source CT severity index was determined by aggregating the scores attributed to inflammatory reactions, necrosis, and extrapancreatic complications. Additionally, the Marshall scoring system, a holistic measure of the severity and prognosis of multi-organ dysfunction syndrome, was adopted to assess the organ function status of patients, capping at a total of 12 points.

### Statistical methods

All quantitative data are delineated as mean ± standard deviation. Paired *t*-tests facilitated the within-group comparisons relative to the baseline values, while independent sample *t*-tests facilitated the analyses of between-group disparities concerning treatment-related variations. The chi-square test was utilized to examine the overall therapeutic efficacy, presuming a normal distribution. A significance level of P < 0.05 was considered statistically significant. After data organization and verification, data analysis was performed using SPSS 23.0 statistical software.

## Results

At the study's conclusion, two individuals from the control group were relocated to a superior-level hospital for advanced care, constituting a slight attrition in the participant pool. Consequently, the analysis incorporated data from 34 cases in the observation group and 32 in the control group, as delineated below.

### Lipid concentrations pre and post treatment across both groups

Prior to the intervention, no significant disparities were detected between the groups regarding the levels of TG, TC, HDL, and LDL (P > 0.05). Following treatment, both contingents exhibited a notable diminution in TG and TC levels (Table 1). A comparative analysis revealed a more substantial decline in the TG and TC levels within the observation group at the 24-h post-treatment mark, attaining statistical significance (P < 0.05). Additionally, the observation group witnessed a significant escalation in HDL levels post-treatment, alongside a considerable decrement in LDL levels both pre and post intervention. Conversely, the control group demonstrated a singular decrease in LDL levels pre-treatment. A pronounced enhancement in HDL was observed 24 h post-treatment in the observation group in comparison to the control group, reaching statistical significance (P < 0.05). Nonetheless, no significant variations in lipid components were noted between the groups prior to discharge (P > 0.05). The data propose that the DFAPP intervention facilitated a swift and substantial reduction in triglycerides within the observation group. The respective t-values and P-values are enumerated as follows: Observation group: Post-treatment TG: t = 8.38, P < 0.0001; Pre-discharge: t = 8.02, P < 0.0001; Post-treatment TC: t = 15.3, P < 0.0001; Pre-discharge: t = 14.14, P < 0.001. Control group: Post-treatment TG: t = 6.47, P < 0.0001; Pre-discharge: t = 5.61, P < 0.0001; Post-treatment TC: t = 9.20, P < 0.001; Pre-discharge: t = 10.80, P < 0.001.

**Table 1:** Comparison of blood lipid levels between two groups of patients before and after treatment (mmol/L ${\overline{\text{X}}} \pm S$).

Group	Sample	TG	TC	HDL	LDL
T					
Pre-intervention	34	29.56 ± 17.85	14.74 ± 3.85	0.78 ± 0.34	4.21 ± 1.85
Post-treatment 24 h		3.75 ± 1.95*	3.82 ± 1.58*	1.92 ± 1.18*	1.62 ± 0.58*
Pre-discharge		4.85 ± 2.35*	4.26 ± 1.95*	1.73 ± 0.58*	1.94 ± 0.83*
C					
Pre-intervention	32	27.82 ± 15.48	13.85 ± 4.18	0.81 ± 0.46	4.04 ± 1.72
Post-treatment 24 h		9.57 ± 3.84*^△^	5.58 ± 2.59*^△^	1.28 ± 0.65^△^	2.52 ± 0.85*
Pre-discharge		6.68 ± 2.65*	4.78 ± 1.94*	1.56 ± 0.67	2.18 ± 0.74*
T value		7.83	5.53	2.07	2.66
P value		< 0.05	0.001	0.02	0.11

*T* treatment group, *C* control group; Compared with pre-intervention, *P < 0.05 within the group; Compared with the control group during the same period, ^△^P < 0.05.

### Cytokine levels before and after treatment across both groups

Prior to the intervention, no discernible differences in cytokine levels (IL-6, IL-17, IL-10, IL-35, TNF-α) between the two groups (P > 0.05). Post-treatment, a significant reduction in the pro-inflammatory cytokines IL-6 and TNF-α was noted in both groups, albeit without significant inter-group variations (Table 2). Notably, IL-17 levels diminished significantly within the observation group, a change not mirrored in the control group. Furthermore, the observation group experienced significant intra-group alterations in anti-inflammatory cytokines IL-10 and IL-35, compared to the control group, with IL-35 recording a more pronounced decrease (P = 0.003). This indicates that DFAPP treatment in the observation group effectively inhibited pro-inflammatory cytokines while enhancing anti-inflammatory cytokine activity, thereby modulating the inflammatory response within the body. The precise numerical comparisons are presented below: IL-6: Within-group comparison, observation group t = 13.24, P < 0.0001; control group t = 14.49, P < 0.0001. Between-group comparison after treatment showed no significant differences (P = 0.56). IL-35: Within-group comparison, observation group t = 6.44, P < 0.0001; control group t = 1.99, P = 0.051. TNF-α: Within-group comparison, observation group t = 19.33, P < 0.000; control group t = 19.65, P < 0.0001. Between-group comparison after treatment showed no significant differences (P = 0.19). IL-10: Within-group comparison, observation group t = 2.12, P = 0.03; control group t = 0.34, P = 0.95. There were no statistically significant differences between the groups after treatment (P > 0.05). IL-17: Within-group comparison, observation group t = 6.32, P < 0.001; control group showed no significant differences in both within-group and between-group comparisons (P > 0.05).

**Table 2:** Comparison of cytokine levels between two groups of patients before and after treatment (${\overline{\text{X}}} \pm S$).

Group	Sample	IL-6 (pg/mL)	IL-17 (ng/mL)	IL-10 (ng/mL)	IL-35 (ng/mL)	TNF-α (ng/mL)
T						
Pre-intervention	34	1.56 ± 0.58	9.85 ± 3.65	45.84 ± 11.85	6.85 ± 2.62	326.45 ± 57.25
Post-treatment 48 h		0.21 ± 0.13*	5.25 ± 2.16*	52.73 ± 14.87*	4.97 ± 2.28*	124.55 ± 20.75*
C						
Pre-intervention	32	1.54 ± 0.49	9.92 ± 3.78	46.75 ± 12.48	6.29 ± 2.96	321.50 ± 52.80
Post-treatment 48 h		0.23 ± 0.15*	6.31 ± 2.85	49.62 ± 11.58	3.52 ± 1.49^△^	131.8 ± 24.50*
T value		0.43	1.25	0.34	3.07	0.98
P value		0.55	0.29	0.94	0.003	0.48

*T* treatment group, *C* control group; Compared with pre-intervention, *P < 0.05 within the group; Compared with the control group during the same period, ^△^P < 0.05.

### Serum biomarker levels before and after treatment in both groups

Prior to the intervention, the serum biomarker concentrations (CRP, PCT, BUN, ALB, WBC) exhibited no significant distinctions between the two groups (P > 0.05). Post-treatment, both groups manifested comparable efficacy in diminishing inflammation (Table 3), albeit the observation group displayed a markedly greater reduction in CRP levels (^△^P < 0.05). Furthermore, the observation group experienced a less pronounced decline in PCT and WBC levels compared to the control group, yet these changes were not statistically significant. Notably, the ALB concentrations in the observation group demonstrated a swifter recuperation post-treatment (^△^P < 0.05). The therapy yielded uniform beneficial impacts on kidney function across both groups. These findings suggest that DFAPP therapy possesses a substantial benefit in mitigating sepsis-related inflammation and enhancing hepatic and renal functions within the observation group. Detailed numerical analyses are presented below: CRP: Within-group comparison, observation group t = 21.89, P < 0.05; control group t = 19.52, P < 0.05. PCT: Within-group comparison, observation group t = 6.53, P < 0.05; control group t = 6.24, P < 0.05. BUN: Within-group comparison, observation group t = 5.0, P < 0.05; control group t = 5.07, P < 0.05. ALB: Within-group comparison, observation group t = 4.78, P < 0.05; control group t = 1.70, P = 0.08. WBC: There were no significant differences between the two groups after treatment (P > 0.05).

**Table 3:** Comparison of biochemical levels between two groups of patients before and after treatment (${\overline{\text{X}}} \pm S$).

Group	Sample	CRP	PCT	BUN (mmol /L)	ALB (g/L)	WBC/(10^9^/L)
T						
Pre-intervention	34	178.28 ± 25.72	3.52 ± 1.83	12.75 ± 3.98	29.58 ± 4.26	18.85 ± 7.52
Post-treatment 72 h		68.67 ± 13.82*	1.22 ± 0.93*	8.72 ± 2.50*	34.12 ± 3.54*	8.92 ± 3.13*
C						
Pre-intervention	32	174.50 ± 32.32	3.62 ± 1.83	12.62 ± 3.87	29.97 ± 4.35	19.21 ± 8.50
Post-treatment 72 h		75.26 ± 16.82*^△^	1.35 ± 0.94*	8.36 ± 2.75*	31.65 ± 3.24^△^	10.32 ± 3.27*
T value		2.01	0.54	0.55	2.7	1.77
P value		0.04	0.57	0.57	0.007	0.08

*T* treatment group, *C* control group; Compared with pre-intervention, *P < 0.05 within the group; Compared with the control group during the same period, ^△^P < 0.05.

### Comparison of Marshall score, APACHE II score, and MCTSI score before and after treatment in both groups

Initially, no significant variations were discernible in the three evaluated scores (Marshall, APACHE II, MCTSI) between the groups (P > 0.05). Post-intervention, the observation group exhibited diminished Marshall scores relative to the control group (Table 4), indicative of superior preservation of organ functionality. Both factions demonstrated efficacious enhancements in APACHE II scores, with no substantial variations observed within a 72-h frame in the pancreatic CT evaluations. Detailed numerical analyses are delineated below: Marshall score: Observation group t = 5.19, P < 0.05; control group t = 2.54, P = 0.01. APACHE II score: Observation group t = 8.50, P < 0.05; control group t = 5.66, P < 0.05. MCTSI: Observation group t = 1.99, P > 0.05; control group t = 1.47, P > 0.05.

**Table 4:** Comparison of Marshall score, APACHE II score, and MCTSI score between two groups of patients before and after treatment (${\overline{\text{X}}} \pm S$).

Group	Sample	Marshall	APACHE II	MCTSI
T				
Pre-intervention	34	4.75 ± 1.45	15.80 ± 3.75	6.15 ± 1.82
Post-treatment 72 h		2.85 ± 1.35*	9.65 ± 1.87*	5.35 ± 1.48
C				
Pre-intervention	32	4.58 ± 1.74	16.15 ± 3.45	6.22 ± 1.80
Post-treatment 72 h		3.56 ± 1.46^△^	11.72 ± 2.78*^△^	5.57 ± 1.73
T value		2.05	3.57	0.56
P value		0.04	0.007	0.58

*T* treatment group, *C* control group; Compared with pre-intervention, *P < 0.05 within the group; Compared with the control group during the same period, ^△^P < 0.05.

### Comparison of hospitalization duration, mortality rate, 6-month recurrence rate, and lipid levels between the two groups

In comparison to the control group, the observation group showcased commendable short-term and long-term outcomes, characterized by reduced average durations of hospital stay, diminished mortality rates, and a lower incidence of pancreatitis recurrence at the 24-week milestone, all of which denoted significant statistical disparities (P < 0.05). Although the observation group recorded lower average TG levels at the 24-week assessment, this discrepancy did not attain statistical significance (P < 0.05), and the results are shown in Table 5.

**Table 5:** Comparison of hospitalization time, mortality rate, 12-week pancreatitis recurrence rate, and blood lipid follow-up between two groups of patients.

Group	Sample	Duration of treatment (days)	Fatality rate (%)	Recurrence rate (%)	TG (mmol/L)
T	34	13.5 ± 2.50	2.90	14.70	3.72 ± 0.84
C	32	17.5 ± 4.50^△^	12.50	25.00^△^	6.68 ± 2.52^△^
t/χ^2^ value		4.49	0.46	3.84	6.48
P value		< 0.05	< 0.05	< 0.05	< 0.05

*T* treatment group, *C* control group; Compared with the control group during the same period, ^△^P < 0.05.

## Discussion

Acute Pancreatitis (AP) is classified into mild AP, moderately severe AP, severe AP, and critical acute pancreatitis (CAP). The causes include cholelithiasis, alcoholism, and hypertriglyceridemia. Literature reports^7,8^ indicate that the mortality rate of hypertriglyceridemia-induced moderately/severe acute pancreatitis (HL-M/SAP) in the past decade has reached 30–42%. The pathogenesis is not yet clear but is closely related to the lipotoxicity of triglycerides, pancreatic microcirculation disorders, and cytokine storm sepsis (CSS).

Reducing triglycerides is an effective method for treating HL-M/SAP. Our study results suggest that three hours after treatment with the blood purification DFAPP mode, patients' triglyceride levels rapidly decreased to near-normal levels (3.75 ± 1.95). This was accompanied by an improvement in the body's inflammatory storm, which might be related to the rapid reduction of triglycerides by DFAPP, alleviating the toxic effects of fatty acids and improving microcirculation. However, the improvement in patient TG levels was relatively poor (9.57 ± 3.84) after treatment with the CVVH + PA purification mode. This was mainly due to the slow clearance of large molecules such as triglycerides by the CVVH + PA purification mode, leading to the chronic persistence of fatty acid toxicity. In physiological conditions, triglycerides account for over 80% of fat cell mass^9^, and obesity leads to fat accumulation around the pancreas and internal organs. Research by Saisho et al. has confirmed that pancreatic fat increases with BMI^10^. When there is an excess of triglycerides (TG) in the body's circulation, a high concentration of TG also accumulates in the pancreatic capillaries. The breakdown of these triglycerides into free fatty acids (FFA) induces glandular cell inflammation, edema, degeneration, necrosis, and other damages^11^. At the same time, the pancreatic microenvironment becomes acidic, activating pancreatic enzymes and leading to autodigestion of the pancreas. Additionally, high concentrations of fatty acids can directly damage the endothelial cells of pancreatic capillaries, causing contraction dysfunction and permeability disorders, blood circulation stagnation, and resulting in pancreatic acinar cell ischemia and hypoxia, inflammation and necrosis, further exacerbating the condition. Therefore, reducing triglycerides as early as possible can not only alleviate lipotoxicity and improve microcirculation but also protect acinar function and halt disease progression.

Reducing triglycerides also alleviates cytokine storms and prevents MODS. Our study found that after DFAPP treatment, the pro-inflammatory cytokines IL-6, IL-17, and TNF-α in the observation group significantly decreased compared to pre-treatment levels. Compared to the control group during the same period, the reduction of pro-inflammatory cytokines was even more pronounced. The control group also showed a decrease in pro-inflammatory cytokines after treatment with CVVH + PA purification, but the effect was not as good as with DFAPP treatment. Persistent systemic inflammation led to slower disease mitigation, thereby prolonging the average hospital stay for the control group. In animal experiments with HTG rats^12^, it was found that with the increase of fatty acid levels, cytokine levels including IL-6, TNF-α, and IL-17 also increased. Fatty acids rich in human necrotic bacteria were found, and acute unsaturated fatty acids produced by lipolysis caused pancreatic necrosis, systemic inflammation, and worsening of SAP-related damage. Inhibiting lipolysis reduced the production of FFA and improved the adverse outcomes of AP. Multiple studies^10,13^ found a close correlation between fat concentration in adjacent acinar tissue and organ damage in AP. Simultaneously, fatty acids in the pancreas had a direct toxic effect on pancreatic tissue during acute pancreatitis. Our study found that the incidence of concurrent MODS events within 48 h after DFAPP treatment was significantly lower than in the control group. This may be due to DFAPP rapidly reducing triglycerides, not only alleviating fatty acid toxicity but also blocking the resulting inflammatory response. At the same time, DFAPP quickly adsorbs and removes cytokines, reducing inflammatory damage and thus protecting organ function. Ultimately, the observation group had better APACHE II scores, suggesting that DFAPP promotes rapid recovery and significantly reduces mortality compared to the control group (2.9% vs 12.5%). A study on markers of severe pancreatitis^14^ found that fatty acids produced by lipolysis around the pancreas can cause more severe cytokine release and multi-organ failure as well as death. It also promotes the progression of mild acute pancreatitis to severe pancreatitis. Clinically, it has been observed that free fatty acids in the serum of patients with severe acute pancreatitis are significantly increased^15^.

Cytokine storm syndrome (CSS) plays a crucial role in the onset and progression of HL-M/SAP, and its initiation is inseparable from fatty acid toxicity^16^. Free fatty acids trigger a variety of cytokines, which mediate an inflammatory storm, leading to persistent organ failure, a primary cause of early mortality in severe acute pancreatitis (SAP). Studies have found that cytokines, particularly TNF-αand IL-6, along with lipid factors such as resistin and visfatin, are significantly elevated in the serum of SAP patients^17–19^. Our study discovered that pro-inflammatory factors such as IL-6, IL-17, TNF-α, CRP, WBC, PCT, etc., significantly increase after the onset of HL-M/SAP. Anti-inflammatory factors like IL-10 also rise. After blood purification treatment, these pro-inflammatory factors, along with triglycerides and cholesterol, decrease. Post-treatment changes in anti-inflammatory factors occur: IL-10 increases compared to pre-treatment levels, IL-35 decreases, but the anti-inflammatory factor IL-35 after DFAPP intervention is higher than the mean value after CVVH + PA intervention. This suggests that DFAPP exhibits a more pronounced effect in inhibiting inflammatory storms. Inflammatory response is a double-edged sword; activation is accompanied by suppression. Clinical research indicates that pro-inflammatory cytokines such as IL-17, IL-6, IL-33, TNF-α, etc., significantly increase in severe acute pancreatitis^20^. Anti-inflammatory cytokines like IL-10, IL-35 receptor antagonists, soluble IL-2 receptor levels are also higher in severe acute pancreatitis than in mild acute pancreatitis^21^, which is consistent with our study results. Animal experiments have found that in obese mice given IL-12, IL-18 or rats infused with free fatty acids in the ducts, the result induced SAP and increased mortality^13,22^. In these models, cytokines (TNF-α, IL-6 and MCP-1) levels in obese mice increased along with serum fatty acids. However, after treatment with the lipase inhibitor Orlistat, these cytokines and fatty acids levels decreased. This indicates that the increase in cytokines is a response to free fatty acids produced by lipolysis. It also suggests that fatty acids can trigger cytokine damage.

In our study, we found that as triglycerides were rapidly cleared in the observation group, the body's inflammatory storm was subsequently suppressed. The Marshall score was lower than the control group; albumin and creatinine levels recovered quickly. The effect of DFAPP treatment on protecting organ function was more apparent. Therefore, early clinical removal of high triglycerides and inflammatory mediators has become the primary goal for treating lipid-induced severe acute pancreatitis complicated by multiple organ dysfunction syndrome^22^. Our study also found that the recurrence rate of pancreatitis in the observation group was 10.3% lower than the control group after a 24-week follow-up. The mean triglyceride level was significantly lower than the control group (3.72 ± 0.84 vs 6.68 ± 2.52). This may be related to the high expression of anti-inflammatory factors IL-10, IL-35 after DFAPP purification, which is beneficial for liver lipid metabolism. At the same time, it may also be related to the rapid decline of triglycerides and cholesterol after DFAPP treatment, causing the restoration of lipid balance due to the increase in high-density lipoproteins and the decrease in low-density lipoproteins.

In the realm of blood purification research related to HL-AP, both domestic and international reports focus on aspects such as plasma exchange, double filtration plasmapheresis, and hemofiltration. The DFAPP model innovatively combines DFPP and CPFA techniques, earning a patent for artificial liver technology (Patent No. 202110671354.9). This study suggests that early use of blood purification through DFAPP treatment can rapidly reduce triglycerides, inhibit cytokine storm syndrome damage, decrease the incidence of multiple organ failure, enhance patient survival rates, and lower the recurrence rate of pancreatitis. In conclusion, the DFAPP blood purification model is worth promoting for clinical use in HL-M/SAP and warrants further in-depth research.

## Data Availability

The original contributions presented in the study are included in the article, further inquiries can be directed to the corresponding author.
